# Brain serotonin and dopamine modulators, perceptual responses and endurance performance during exercise in the heat following creatine supplementation

**DOI:** 10.1186/1550-2783-5-14

**Published:** 2008-09-30

**Authors:** Marios Hadjicharalambous, Liam P Kilduff, Yannis P Pitsiladis

**Affiliations:** 1 Department of Life & Health Sciences, University of Nicosia, Nicosia, Cyprus; 2 Integrative and Systems Biology, Faculty of Biomedical and Life Sciences (IBLS), University of Glasgow, Glasgow, UK; 3 School of Human Sciences, University of Wales, Swansea, UK

## Abstract

**Background:**

The present experiment examined the responses of peripheral modulators and indices of brain serotonin (5-HT) and dopamine (DA) function and their association with perception of effort during prolonged exercise in the heat after creatine (Cr) supplementation.

**Methods:**

Twenty one endurance-trained males performed, in a double-blind fashion, two constant-load exercise tests to exhaustion at 63 ± 5% $\dot{\text{V}}$O_2 max _in the heat (ambient temperature: 30.3 ± 0.5 °C, relative humidity: 70 ± 2%) before and after 7 days of Cr (20 g·d^-1 ^Cr + 140 g·d^-1 ^glucose polymer) or placebo (Plc) (160 g·d^-1 ^glucose polymer) supplementation.

**Results:**

3-way interaction has shown that Cr supplementation reduced rectal temperature, heart rate, ratings of perceived leg fatigue (*P *< 0.05), plasma free-tryptophan (Trp) (*P *< 0.01) and free-Trp:tyrosine ratio (*P *< 0.01) but did not influence the ratio of free-Trp:large neutral amino acids or contribute in improving endurance performance (Plc group, n = 10: 50.4 ± 8.4 min vs. 51.2 ± 8.0 min, *P *> 0.05; Cr group, n = 11: 47.0 ± 4.7 min vs. 49.7 ± 7.5 min, *P *> 0.05). However, after dividing the participants into "responders" and "non-responders" to Cr, based on their intramuscular Cr uptake, performance was higher in the "responders" relative to "non-responders" group (51.7 ± 7.4 min vs.47.3 ± 4.9 min, *p *< 0.05).

**Conclusion:**

although Cr influenced key modulators of brain 5-HT and DA function and reduced various thermophysiological parameters which all may have contributed to the reduced effort perception during exercise in the heat, performance was improved only in the "responders" to Cr supplementation. The present results may also suggest the demanding of the pre-experimental identification of the participants into "responders" and "non-responders" to Cr supplementation before performing the main experimentation. Otherwise, the possibility of the type II error may be enhanced.

## Background

It is well established that perception of effort is elevated and exercise performance is markedly impaired in hot environments but the precise mechanism(s) of fatigue have yet to be determined [1]. The previous observation of the maintenance of carbohydrate reserves at exhaustion [2] in conjunction with no impairment in the capacity of skeletal muscle to generate force during exercise with hyperthermia [3] may preclude peripheral factors as the main cause of fatigue during exercise in the heat. Previously, Nielsen and colleagues [4] proposed a core temperature increase to approximately 39.6°C as a critical factor in the reduction of central neural motivation and exercise performance. In subsequent studies, it was proposed that fatigue during exercise in the heat may occur at a critical brain temperature, through a reduction in cerebral blood flow [5,6] and brain glucose levels [7,8]. However, the exact mechanism(s) for these hyperthermia-induced effects and/or how thermal stress may affect brain neurotransmission during exercise in the heat are presently unknown.

Several studies have attributed the changes in body temperature [e.g., [9,10]], the higher effort perception and impaired exercise tolerance during exercise in the heat to events localized within the central nervous system, and in particular, the serotonergic and dopaminergic systems [11-13]. Soares et al. [12,13] for example, suggested that core body temperature was elevated and exercise performance was diminished following pre-exercise intracerebroventicular injection of tryptohan (Trp: i.e. a 5-HT precursor) in rats. Lin et al. [10] also observed that when hypothalamic serotonin (5-HT) levels were increased in the rat by administration of Fluoxetine (i.e. 5-HT reuptake inhibitor) and 5-hydoxytryptophan (i.e. a 5-HT precursor) there was an increase in metabolic heat production with a concomitant reduction in heat loss. In addition, Bridge et al. [14], using a combined Buspirone (i.e. 5-HT_1A _agonists/dopamine D_2 _antagonist) and Pindolol (5-HT_1A _antagonist) neuroendocrine challenge, found the dopaminergic-induced prolactin (Prl) response to be significantly correlated both with submaximal exercise duration at 35°C, rectal temperature and the rate of temperature rise. They concluded that high activity of the dopaminergic pathways in the hypothalamus was a predictor of exercise tolerance in the heat. More recently, Watson et al., [15] examined the effect of the dual dopamine/noradrenaline reuptake inhibitor (bupropion) on performance, thermoregulation and hormonal responses during exercise in the heat (30°C) and in temperate (18°C) conditions. They found that although perception of effort and thermal stress were not different between bupropion and placebo trails during exercise in the heat, exercise performance was enhanced by acute bupropion administration. No such effect was seen at temperate conditions. These authors suggested that bupropion may diminish inhibitory signals arising from the CNS to discontinue exercise due to hyperthermia enabling participants to increase performance.

Creatine (Cr) is abundant in muscles and the brain and after phosphorylation used as an energy source for adenosine triphosphate synthesis [16]. Cr for example, was found to improve performance during high intensity exercise by increasing intramuscular Cr and phosphocreatine (PCr) levels [17] and by accelerating the resynthesis rate of PCr [18]. However, although the role of Cr in protecting muscle fatigue during high intensity exercise was extensively studied and understood, its role in preventing thermal-stress and central fatigue developments was not. Cr for example, has been shown to elevate total body water (TBW) [19,20], enhance body thermal tolerance, reduce core temperature and heart rate and improve performance during exercise in the heat [21]. However, whether these effects of Cr on thermoregulation and exercise performance were due to better maintenance of increase TBW or central neural effects is presently unknown. It was found for example, that oral Cr supplementation improved mental function and reduced mental fatigue by increasing the oxygen utilization in the brain [16]. In addition, several pharmacological studies suggested that oral Cr supplementation has been found to increase brain DA synthesis in the substantia nigra of mice by protecting against striatal dopamine depletion [22] and/or by enhancing tyrosine hydroxylase activation (the rate-limiting enzyme of brain DA biosynthesis) [23,24]. However, no studies were examined so far the responses of peripheral modulators and indices of brain 5-HT and DA function during exercise in the heat after Cr supplementation, The purpose therefore of the present experiment was to examine the effects of oral Cr, used as a physiological model to enhance body thermal tolerance, on perceptual responses and on central fatigue development by measuring peripheral modulators and indices of brain 5-HT and DA metabolic interaction during exercise in the heat in trained humans.

## Methods

### Participants

Twenty one endurance-trained males volunteers (Table 1) provided written informed consent for the study, which was approved by the Glasgow University Research Ethical Committee. Participants were recruited from local athletics and cycling clubs and none were acclimatized to exercise in the heat. Participants eligibility was initially assessed by interview. No participant had a history of cardiovascular or respiratory disease and/or evidence of musculoskeletal injury. All participants were Cr free for at least 8 weeks prior to the study. The investigators did not reveal prior to interview that participants would be excluded if they had supplemented with Cr in the 8 weeks preceding the study. One participant from the placebo (Plc) group had previously supplemented with Cr. No Cr was detected in the baseline urine samples of any participant.

**Table 1:** Physical characteristics of the two groups of participants. Values are presented as the mean ± SD

	**Placebo Group (n = 10)**		**Creatine Group (n = 11)**	
	**Pre**	**Post**	**Pre**	**Post**
Age (yr)	27 ± 4	-	27 ± 5	-
Height (cm)	181 ± 4	-	178 ± 7	-
Weight (kg)	71.0 ± 6.0	71.2 ± 6.0	72.7 ± 6.6	73.4 ± 6.6*
Total body water (L)	40.4 ± 3.3	40.5 ± 3.2	41.0 ± 3.1	41.6 ± 3.2*
Intracellular Water (L)	21.9 ± 1.8	21.9 ± 1.8	22.2 ± 1.8	22.7 ±
Extracellular Water (L)	18.5 ± 1.5	18.5 ± 1.4	18.8 ± 1.4	18.9 ± 1.5
$\dot{\text{V}}$O_2 max _(L·min^-1^)	4.3 ± 0.4	-	4.5 ± 0.4	-
$\dot{\text{V}}$O_2 max _(ml·kg·min^-1^)	60.5 ± 4.7	-	61.4 ± 4.6	-
Max work rate (watts)	350 ± 34	-	373 ± 31	-

* Indicates a significant difference from pre-supplementation values

### Experimental design

Participants initially underwent a continuous incremental test to volitional exhaustion in order to determine the lactate threshold (LT), maximum oxygen consumption ($\dot{\text{V}}$O_2 max_) and the maximal work rate (WR_max_). Following the maximal incremental exercise test, participants visited the laboratory on at least two separate occasions in order to become familiar with the exercise protocol and experimental procedures, in addition to establishing a suitable work rate (WR) that would elicit fatigue in 40–60 min. This was achieved by setting the WR at 20% Δ (i.e., 20% of the difference between the $\dot{\text{V}}$O_2 _at the LT and $\dot{\text{V}}$O_2 max_) during the initial familiarization session and, where necessary, adjusting the WR for subsequent trials to achieve the desired duration. This intensity of exercise was chosen to avoid fatigue occurring as a result of muscle glycogen depletion [2]. Following the familiarization period (at least two familiarization trails were carried out identical to the experimental trails with exception of complete blood collections), participants were matched for body mass and were randomized, in a double-blind fashion, to receive either Cr or Plc trials. Participants performed one constant-load exercise test to volitional exhaustion pre-supplementation and one post-supplementation. The first test was conducted at least 48 hrs after the final familiarization trial. The supplementation period for both groups started on the day after the first test and finished the day before the second test.

### Supplementation protocol

Cr supplementation (Creatamax 300, Maximuscle Ltd., Watford, UK) consisted of 22.8 g·d^-1 ^Cr·H_2_O (equivalent to 5 g Cr × 4 daily) and 35 g of glucose polymer (Maxim, Geffen, Holland) made up in 500 mls of warm to hot water for 7 days taken at equal intervals throughout the day. This protocol has been shown to increase resting muscle PCr levels within 5 days [17]. Each supplementation was freshly made prior to consumption in order to prevent any degredation of Cr to creatinine (Crn). The Plc group consumed 160 g·d^-1 ^of glucose polymer (40 g × 4 daily) for 7 days, prepared and administered in an identical fashion to the Cr supplement. Both supplements had similar taste, texture and appearance and were placed in generic packets to ensure double-blind administration. Participants otherwise followed their normal diet but eliminated caffeine and caffeine-containing foods throughout the experimental period to minimize the possible inhibitory effects of caffeine on the ergogenic effect of Cr. At the end of the study all participants gave verbal assurance that they had complied with these instructions.

### Procedures

All exercise tests were carried out between 18:00 and 20:00 hr. Participants reported to the laboratory on the day of testing after a standardized meal and having refrained from alcohol and strenuous exercise the day before. The participant's left hand and forearm were immersed in water at 42–44°C for fifteen minutes in order to allow for arterialization of the venous blood [25]. Following this, a 21 G cannula was introduced into a superficial vein on the dorsal surface of the heated hand and a resting blood sample (10 ml) obtained. The venous cannula was kept patent by a slow (c. 0.5 ml·min^-1^) infusion of isotonic saline between samples. The participant was transferred to the climatic chamber (ambient temperature of 30.3 ± 0.5°C with a relative humidity of 70 ± 2% and air velocity of approximately 3.6 m·sec^-1^) and remained seated on the cycle ergometer for a further 5 min. Participants were then instructed to begin 5 min of unloaded cycling before another blood sample was obtained. After 5 min of unloaded cycling, the WR was increased in a "single step" to the predetermined WR and participants maintained a pedal cadence of 60–90 rpm throughout the test. Participants exercised at the same WR for both experiments (i.e., 16 ± 11% Δ or 63 ± 5% O_2 _max, 225 ± 26 watts). Exhaustion was defined as the point at which the participant could no longer maintain the pedal cadence above 60 rpm for a second occasion (at around 15 seconds) after an initial verbal warning from the investigators. Blood samples were obtained at 5 min intervals throughout exercise and at exhaustion. Time to exhaustion was recorded but withheld from the participant until all exercise tests had been completed.

### Blood treatment and analysis

Blood (10 ml) was drawn into dry syringes and dispensed into tubes containing K_3_EDTA and the remainder into tubes containing no anticoagulant. Duplicate aliquots (400 *μ*L) of whole blood from the K_3_EDTA tube were rapidly deproteinised in 800 *μ*L of ice cold 0.3 mol·L^-1 ^perchloric acid. After centrifugation, the supernatant was used for the measurement of glucose and lactate using standard enzymatic methods with spectrophotometric detection (Mira Plus, ABX Diagnostics, Montpellier, France). Some of the uncoagulated blood was also used for the measurement of haemoglobin (Hb) (cyanmethaemoglobin method, Sigma Chemical Company Ltd., Dorset, UK) and packed cell volume (PCV) (conventional microhematocrit (Hct) method). All blood analyses were carried out in duplicate with the exception of PCV, which was analysed in triplicate. Plasma volume changes were calculated from changes in Hb and PCV relative to initial baseline values [26]. An aliquot of whole blood from the K_3_EDTA tubes was centrifuged and the plasma obtained was separated and used for the measurement of free fatty acids (FFA) (colorimetric method, Roche Diagnostics GmbH, Germany) and concentrations of amino acids including total and free Trp, tyrosine (Tyr) and large neutral amino acids (LNAA) by HPLC using fluorescence detection and pre-column derivitisation with 18 *o*-phthalaldehyde (Hypersil Amino acid method, ThermoHypersil-Keystone, Runcorn, UK). The LNAA includes Tyr, phenylalanine, leucine, isoleucine and valine. Free-Trp was separated from protein-bound Trp by filtering plasma through 10,000 NMWL 'nominal molecular weight limit' cellulose filters (Ultrafree MC filters, Millipore Corporation, USA) during centrifugation at 5000 g for 60 min at 4°C. Prior to centrifugation, filters were filled with a 95% O_2 _– 5% CO_2 _mixture in order to stabilize pH. The blood in tubes without anticoagulant was allowed to clot and then centrifuged; the serum collected was used for the measurement of prolactin (Prl) by sandwich magnetic separation assay (Technicon Immuno 1 System, Bayer Diagnostics, Newbury, UK).

### Calculations

The thermal gradient was calculated as rectal temperature (T_rec_) – skin temperature (T_sk_). The calculation of weighted mean T_sk _[T_sk _= 0.3 (T_chest _+ T_arm_) + 0.2 (T_thigh _+ T_calf_)] was according to the method of Ramanathan [27]. Mean body temperature (T_b_) was calculated at each time point as 0.87T_rec_+0.13T_sk _[28].

### Estimated Cr uptake

Participants completed 8 separate 24 hr urine collections. The collection began on the day preceding supplementation (baseline), and then continued through the 7 days of supplementation. The urine volume for each 24 hr period was measured and mixed thoroughly, with a representative 20 mL sample being stored at -20°C for subsequent analysis (ABX Mira Plus Spectrophotometer, ABX Diagnostics, UK) of Cr and Crn concentration, using a spectrophotometric enzymatic Crn Kit (MPR1 – Kit no. 839434, Roche Diagnostics Ltd., East Sussex, UK). Estimated Cr uptake was calculated by subtracting the total Cr excreted, corrected for Crn excretion, from the total amount supplemented per day. Estimated intramuscular [Cr] (mmol·kg^-1^·dry weight muscle) was calculated based on an estimated muscle mass amounting to 40% of body mass and average muscle water approximating 77% of wet weight [29].

### Statistical analysis

Data were expressed as the mean ± SD following a test for the normality of distribution. For data that violated the assumptions for parametric analyses (i.e. equality of variance and normality of distribution) non-parametric analyses was carried out and these data were expressed as the median (Interquartile range: IQR). Statistical analysis was carried out using a mixed 3-way ANOVA (Group × Pre- and Post- supplementation × Time) with repeated measures on the last two factors. A subsequent 2-way ANOVA with repeated measures was performed when there was a main effect on Group, Pre-Post supplementation, or interaction. Two-sample *t*-test (between treatment effect, i.e., magnitude of change (Δ) in the Cr group vs. Δ in the Plc group) and student *t*-test (within treatment effect, i.e., Pre- vs. Post-supplementation) were performed if a main treatment or interaction effect was observed. For non-parametric data, Friedman two-way ANOVA (followed by Wilcoxon test) and Mann-Whitney tests were used for paired and unpaired data, respectively. Pearson product moment r and Spearman rho correlation analyses for parametric and non-parametric data respectively were used to assess the relationship between selected variables. Correlation analysis was performed for each time point separately. Statistical significance was a priori at *p *< 0.05.

## Results

### Physiological responses, anthropometrics and performance data

**Figure 1:**
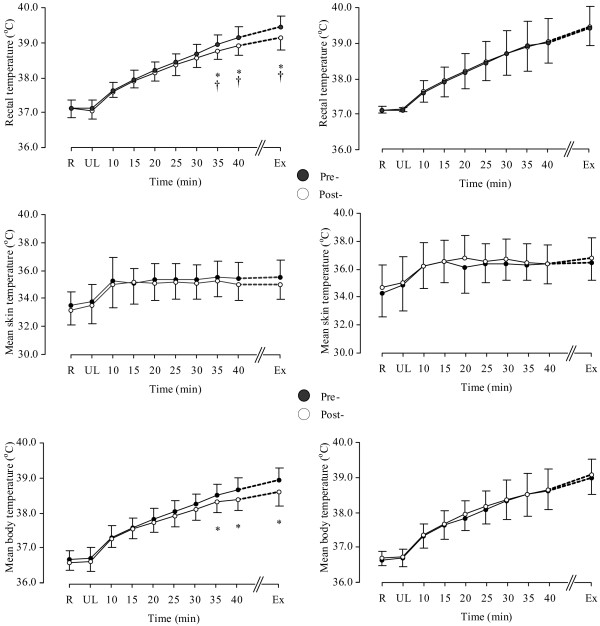
**Rectal temperature (top panel), mean skin temperature (middle panel) and mean body temperature (bottom panel) in the Cr (left side) and placebo (right side) supplemented groups.** *: indicates a significant difference between pre (●) to post (○) supplementation. †: indicates a significant greater change in the Cr group compared with the placebo group (p < 0.05). Values are given as mean (SD).

**Figure 2:**
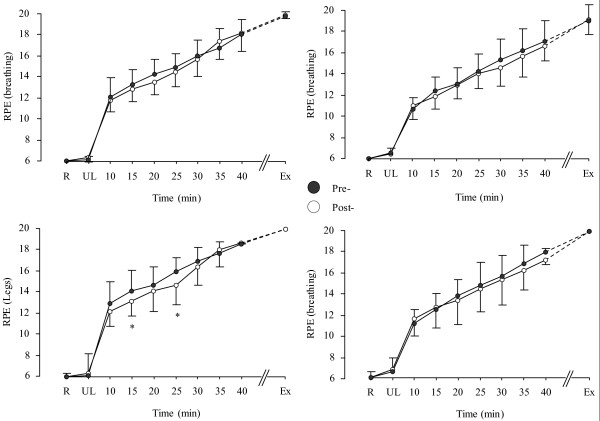
**RPE (breathing) (top panel) and RPE (legs) (bottom panel) in the Cr (left side) and placebo (right side) supplemented groups during exercise.** *: indicates a significant difference between pre (●) to post (○) supplementation. Values are given as mean (SD).

Relative to the Plc group, Cr supplementation increased intracellular water (ICW), TBW and body mass (Table 1) and reduced rectal temperature (T_rec_), mean body temperature (T_b_) (Figure 1), heart rate (HR), sweat rate and ratings of perceived leg fatigue (Figure 2) (3-way interaction; p < 0.05). However, Cr did not influence skin temperature (T_skin_) (Figure 1), total sweat loss, changes in plasma volume, blood [glucose] and [lactate], $\dot{\text{V}}$O_2_, carbon dioxide production ($\dot{\text{V}}$CO_2_), respiratory exchange ratio (RER), minute ventilation ($\dot{\text{V}}$E) and perception of breathlessness (Figure 2). Endurance performance was not different between and within Plc group and Cr groups, as a whole (Plc: 50.4 ± 8.4 min vs. 51.2 ± 8.0 min, p < 0.05; Cr group: 47.0 ± 4.7 min vs. 49.7 ± 7.5 min, p > 0.05). However, after dividing the participants into "responders" and "non-responders" to Cr supplementation based on their intramuscular Cr uptake [29,30], performance increased in the "responders" relative to "non-responders" to Cr group (51.7 ± 7.4 min vs.47.3 ± 4.9 min, *p *< 0.05). Five out of the eleven participants in the Cr group reported that they found the post-supplementation trial easier, while two out of the ten participants in the Plc group rated the post-supplementation trial to be easier. No side effects were reported following Cr and/or Plc supplementations.

### Estimated Cr uptake

Estimated Cr uptake was calculated by subtracting the total Cr excreted, corrected for the increase in Crn excretion, from the total amount supplemented per day. Estimated intramuscular Cr (mmol·kg^-1^·dry weight muscle) was calculated based on an estimated muscle mass amounting to 40% of body mass and average muscle water approximating 77% of wet weight [31]. In the Cr group, Crn excretion increased from 1.4 ± 0.4 g·day^-1 ^pre-supplementation to 2.4 ± 1.0 g·day^-1 ^on the final day of supplementation. There was no increase in Crn excretion in the placebo group (1.5 ± 0.4 g·day^-1 ^to 1.4 ± 0.4 g·day^-1^). Cr excretion increased from 8.7 ± 3.7 g·day^-1 ^pre-supplementation to 17.4 ± 1.9 g·day^-1^; no Cr was detected in the urine of the placebo group. Estimated Cr uptake was maximal on the first day of Cr supplementation [10.4 ± 3.7 g, 56 ± 18% being retained] and was lowest on the final day [2.9 ± 1.9 g, 13 ± 9% being retained]. The total amount of Cr retained over the supplementation period was 39 ± 14 g, with an estimated increase in intramuscular Cr of 45 ± 15 mmol·kg^-1^·dry weight muscle. Based on these estimates, 3 participants were classified as "non-responders" [22 ± 3 mmol·kg^-1^·dry weight muscle] and the remaining 8 participants were classified as "responders" (53 ± 5 mmol·kg^-1^·dry weight muscle) as previously described [29].

### Plasma amino acids, prolactin and free fatty acids

There were no significant differences between Plc and Cr groups in plasma total [Trp], [Tyr], [large neutral amino acids] (LNAA) (Table 2), total [Trp]:[LNAA] ratio, total [Trp]:[Tyr] ratio, free-[Trp]:LNAA ratio and free-[Trp]:[Tyr] ratio (Table 3). The Δ plasma total [Trp], [Tyr] and [LNAA] and the ratios of total [Trp]:[LNAA], total [Trp]:[Tyr] and free-[Trp]:[LNAA] were also not different between the groups. However, plasma free-[Trp] was significantly lower in the post-Cr supplementation trial (χ^2 ^= 33.909, df = 5, p < 0.01) and in the "responders" (χ^2 ^= 25.786, df = 5, p < 0.01) when compared to the pre-supplementation trial (Table 2). The Δ free-[Trp] was also significantly lower during exercise in the Cr group (χ^2 ^= 15.881, df = 1, p < 0.05) and in the "responders" (χ^2 ^= 14.582, df = 1, p < 0.05) when compared with Δ Plc group. In "responders", the plasma free-[Trp]:[Tyr] ratio was significantly lower at rest and during exercise post-Cr, relative to the pre-supplementation trial, (χ^2 ^= 13.071, df = 5, p < 0.05) (Table 3). In addition, there was a tendency for Δ free-[Trp]:[Tyr] ratio to be significantly lower in the "responders" group when compared with Plc group (*p *= 0.071). [LNAA] (Table 2), free-[Trp]:[LNAA] ratio, total [Trp]:[LNAA] ratio, and total [Trp]:[Tyr] ratio (Table 3) were not different between pre- and post-supplementation trials in both Plc and Cr groups as well as in the "responders". Plasma [Prl] was not different between the Plc group and the Cr group as a whole and between pre- and post-supplementation trials in both Plc and Cr groups (Table 4). In all trials, [Prl] increased significantly over time when compared to resting levels. The Δ plasma [Prl] was also not different between the groups. Plasma [FFA] was not different between Plc and Cr groups or between pre- and post-Cr supplementation trials (Table 4).

**Table 2:** Concentrations of total Trp, Tyr, LNAA and free-Trp before and after supplementation.

			**Blood collection time (min)**		
	**Group**	**Trial**	**Rest**	**40**	**End**
Total [Trp] (μmol·l^-1^)	Plc	Pre	58.3(12)	56.7(22)	63.9(11)
		Post	55.2(14)	67.7(22)	75.5(16)^§^
	Cr	Pre	47.8(14)	71.1(16)^§^	69.9(17)^§^
		Post	53.4(5)	62.3(7)^§^	71.7(21)^§^
	Resp	Pre	47.5(6)	65.8(15)^§^	68.7(18)^§^
		Post	53.6(7)	62.4(8)^§^	71.6(20)^§^
					
[Tyr] (μmol·l^-1^)	Plc	Pre	85.3(18)	94.8(12)	116(24)^§^
		Post	76.9(41)	86.5(33)	104.1(36)
	Cr	Pre	71.9(37)	111.5(41)^§^	121.7(46)^§^
		Post	81.3(34)	104.9(31)^§^	112.4(52)^§^
	Resp	Pre	70.3(31)	112.1(36)^§^	118.3(45)^§^
		Post	74.6(33)	109.8(31)^§^	114.5(51)
					
[LNAA] (μmol·l^-1^)	Plc	Pre	831(29)	902(211)	986(254)
		Post	873(233)	897(171)	994(375)
	Cr	Pre	744(334)	1076(340)	945(326)
		Post	812(189)	806(372)	933(361)
	Resp	Pre	732(279)	1051(273)	962(267)
		Post	764(187)	864(473)	871(322)
					
Free-[Trp] (μmol·l^-1^)	Plc	Pre	2.5(0.6)	3.1(0.7)^§^	3.3(0.8)^§^
		Post	2.6(1.2)	3.2(0.7)^§^	3.6(0.9)^§^
	Cr	Pre	2.4(0.7)	2.9(0.6)^§^	3.5(0.5)^§^
		Post	2.4(0.5)	2.6(0.5)*^§^	3.0(0.7)*^§^
	Resp	Pre	2.6(0.9)	3.1(0.6)	3.4(0.6)^§^
		Post	2.3(0.7)	2.7(0.6)*	3.0(0.5)*^§^

Values are given as median (IQR).

* Indicates a significant difference from pre-supplementation values

^§ ^Indicates a significant difference over time compared with the resting value

Resp: "responders"

**Table 3:** Total Trp:LNAA, total Trp:Tyr, free-Trp:LNAA and free-Trp:Tyr ratios before and after supplementation.

			**Blood collection time (min)**		
	**Group**	**Trial**	**Rest**	**40**	**End**
Total [Trp]:[LNAA] ratio	Plc	Pre	.069(.016)	.071(.015)	.076(.016)
		Post	.070(.012)	.072(.017)	.084(.022)
	Cr	Pre	.062(.013)	.069(.010)	.075(.012)
		Post	.073(.016)	.080(.016)	.082(.007)^§^
	Resp	Pre	.062(.012)	.069(.010)	.076(.014)
		Post	.076(.016)	.077(.019)	.082(.006)^§^
					
Total [Trp]:[Tyr] ratio	Plc	Pre	0.65(.24)	0.61(.09)	0.58(.17)
		Post	0.71(.07)	0.68(.18)	0.74(.19)
	Cr	Pre	0.64(.14)	0.63(.07)	0.61(.15)
		Post	0.65(.16)	0.62(.14)	0.65(.09)
	Resp	Pre	0.66(.20)	0.62(.09)	0.61(.17)
		Post	0.71(.16)	0.62(.20)	0.62(.09)
					
Free-[Trp]:[LNAA] ratio	Plc	Pre	.0030(.0007)	.0040(.0010)	.0036(.0020)
		Post	.0031(.0024)	.0038(.0014)	.0048(.0012)
	Cr	Pre	.0034(.0021)	.0032(.0015)	.0037(.0016)
		Post	.0028(.0015)	.0038(.0017)	.0028(.0027)
	Resp	Pre	.0042(.0020)	.0034(.0010)	.0045(.0012)
		Post	.0027(.0015)	.0039(.0017)	.0043(.0025)
					
Free-[Trp]:[Tyr] ratio	Plc	Pre	.032(.017)	.035(.008)	.027(.014)
		Post	.035(.018)	.039(.012)	.043(.013)
	Cr	Pre	.034(.027)	.032(.013)	.027(.012)
		Post	.028(.016)	.028(.013)	.024(.019)
	Resp	Pre	.042(.025)	.033(.011)	.032(.013)
		Post	.032(.017)*	.026(.015)*^§^	.029(.018)

Values are given as median (IQR).

* Indicates a significant difference from pre-supplementation values

^§ ^Indicates a significant difference over time compared with the resting value

Resp: "responders"

**Table 4:** Concentrations of plasma prolactin and FFA before and after supplementation.

			**Blood collection time (min)**		
	**Group**	**Trial**	**Rest**	**40**	**End**
Plasma [Prolactin] (nmol·l^-1^)	Plc	Pre	0.13(.04)	0.51(.38)^§^	0.94(.83)^§^
		Post	0.14(.06)	0.53(.24)^§^	0.93(.14)^§^
	Cr	Pre	0.15(.07)	0.84(.58)^§^	1.07(.63)^§^
		Post	0.15(.05)	0.79(.51)^§^	1.14(.56)^§^
	Resp	Pre	0.15(.04)	1.14(.39)^§^	1.27(.44)^§^
		Post	0.14(.04)	0.98(.46)^§^	1.39(.36)^§^
					
Plasma [FFA] (mmol·l^-1^)	Plc	Pre	0.53(0.47)	0.37(0.18)	0.40(0.34)
		Post	0.38(0.10)	0.28(0.14)	0.23(0.19)
	Cr	Pre	0.42(0.26)	0.29(0.11)	0.31(0.18)
		Post	0.48(0.27)	0.32(0.14)	0.34(0.18)
	Resp	Pre	0.48(0.24)	0.28(0.10)	0.30(0.15)
		Post	0.42(0.20)	0.29(0.10)	0.32(0.16)

Values are given as median (IQR).

^§ ^Indicates a significant difference over time compared with the resting value

Resp: "responders".

### Correlation analysis

There were no significant correlations between plasma free-[Trp] and T_rec_, between free-[Trp] and RPE, between free-[Trp] and plasma [FFA], between T_rec _and [Prl], and between free-[Trp] and [Prl], when examined for each time-point separately.

## Discussion

It has been previously shown that Cr increased water retention [20], enhanced body thermal tolerance [21] and improved endurance performance during exercise in the heat [32] but the mechanism of these Cr-induced effects have not been extensively studied. In the present experiment Cr was used as a "vehicle" in an attempt to reduce thermal stress and examine the responses of peripheral modulators and indices of brain 5-HT and DA function, in association with effort perception during exercise in the heat. The significant observations of the present experiment were the effectiveness of Cr to reduce perception of leg muscular fatigue and lower plasma free-[Trp] and free-[Trp]:[Tyr] ratio. In addition, although these responses have not contributed in enhancing endurance performance in the whole Cr group, after distinguishing the participants into "responders" and "non-responders" to Cr based on their intramuscular Cr uptake [29] performance was improved in the "responders" to Cr group (51.7 ± 7.4 min vs. 47.3 ± 4.9 min, *p *= 0.031). The observation also that five out of the eight "responders" reported that they found the post-supplementation trial to be easier and that these same five participants showed the largest estimated Cr uptake and performance gains may be a further indication of the demanding of separating the participants into "responders" and "non-responders" to Cr supplementation.

Previous studies have attributed the changes in body temperature [9,10], the higher effort perception and impaired endurance performance during exercise in the heat to events localized within the serotonergic and dopaminergic systems [11-14]. Recently, for example, 5-HT precursor drugs were found to elevate body heat storage by increasing metabolic heat production with a concomitant reduction in heat loss [10], and subsequently to reduce exercise performance in the heat [12]. On the other hand, high hypothalamic dopaminergic activity has been shown to be a reasonable predictor of exercise tolerance during prolonged submaximal exercise in the heat [14]. In the present experiment, participants who had supplemented with Cr reported significantly lower thermal stress and ratings of perceived leg fatigue. These may suggest that they were able to discern the benefit of this putative thermal-stress reduction strategy.

The increased hydration status, observed in the present study, seems to be a reasonable contributor to the reduction in effort perception during exercise in the heat following Cr supplementation. However, the similar plasma volume and total sweat rate results in association with no differences in blood metabolites and cardiorespiratory responses observed between trials may augment the possibility that the reduction in participantive fatigue is partially due to the lowered plasma free-[Trp] observed in the Cr group and subsequently to the lower brain 5:HT synthesis. It has been suggested, for example, that a high brain [5-HT]:[DA] ratio increases effort perception (i.e. central fatigue) during prolonged exercise while a low [5-HT]:[DA] ratio may favour increased arousal and central neural motivation [33]. The role of DA, in protecting against central fatigue development, has also recently been supported by Watson et al. [15] who found that a dual dopamine/noradrenaline reuptake inhibitor, enhanced exercise performance in the heat but not in temperate conditions. In addition, it has been shown that DA microinjections into the hypothalamus and substantia nigra of the rat produced hypothermia through an increase in brain [DA] [34,35]. This notion was recently supported by Lieberman et al. [36] who observed that L-Tyr supplementation, used as the primary brain catecholamine precursor, was able to increase brain DA and non-epinephrine levels contributing to the reduction in body heat-storage in rats that were exposed in hot environment (41°C).

Nybo et al. [8] however, found that brain uptake of Trp and Tyr as well as brain DA release were not affected by hyperthermia, failing therefore to support the classic "5-HT-central fatigue hypothesis" [37]; and also the involvement of brain DA function in thermoregulation during exercise in the heat. They suggested that brain glycogen depletion may have contributed to central fatigue during exercise in the heat. However, the results presented by these authors do not preclude the involvement of modulators of brain 5-HT function in central fatigue since a correlation was found between arterial free-[Trp] and brain Trp uptake [8]. Brain Trp uptake for example, has been shown on numerous occasions to be the rate-limiting step of 5-HT synthesis [38], thus an involvement of 5-HT during exercise in the heat as classically proposed cannot be excluded. It should be noted that brain 5-HT was not measured in the study by Nybo et al. [8]. Consequently, the finding of a lower plasma free-Trp:Tyr ratio (and by extrapolation a lower 5-HT:DA ratio since both precursors share the same L-system transport across the blood brain barrier [39]) may have contributed to reducing thermal stress and to attenuating effort perception following Cr supplementation. It is noted that no such response was observed in Plc trials.

On the other hand, it seems to be unreasonable to suggest that the lower plasma free-[Trp] and free-Trp:Tyr ratio may have contributed to the improvement in performance in the "responders" to Cr group. For the reason that plasma free-[Trp] was lower in both "responders" and "non-responders" but performance was improved only in the "responders" to Cr group (Table 2). Similarly although, the plasma free-Trp:Tyr ratio was lower in the "responders" group this was evident only until the 40 min time-point and not at exhaustion. It is possible therefore that Cr is effective in reducing effort perception, through to a lower plasma free-[Trp], during exercise in the heat in both "responders" and "non-responders" to Cr loading; however, for eliciting an endurance performance improvement different but still unknown mechanism(s) may play a role. In the current investigation and in most of the related literature involving humans, only indirect markers of brain 5-HT and DA function are assessed. Nevertheless, several pharmacological studies, suggested that oral Cr supplementation increased brain DA synthesis in the substantia nigra of mice by protecting against striatal dopamine depletion [22] and/or by enhancing tyrosine hydroxylase activation (the rate-limiting enzyme of brain DA biosynthesis) [23,24]. In addition, an improvement in mental function and diminished central fatigue was observed during performing a mathematical calculation following oral Cr supplementation [16]. Consequently, more controlled studies are warranted to examine the potential effect of Cr on central fatigue during exercise in the heat.

Plasma [Prl] was found to be similar in all trials; however, since no differences observed in plasma [LNAA] and in the ratio of free and total Trp:LNAA the similar plasma [Prl] results and the lack of significant correlation between plasma [Prl] and T_rec _were not entirely unexpected. According to Wurtman [40] for example in order to elicit a reduction in brain Trp uptake and, therefore, attenuation in brain 5-HT turnover, a 5- to 6-fold elevation in plasma [LNAA] is required. In addition, Leathwood and Fernstrom [41] suggested that a 13- to 26- fold elevation in total Trp:LNAA ratio is required to induce a change in the brain stem [5-HT] in monkeys. The association however, between peripheral modulators of brain 5-HT function and circulating [Prl] during exercise has not been fully explained. Fischer et al. [42] for example, observed an increase in [Prl] during exercise in proportion to the rise in plasma free-[Trp]. Some investigators found a positive correlation between serum [Prl] and T_rec _[43,44] during exercise in the heat and this relationship has been used to justify brain monoamine system involvement in regulating T_rec _and Prl secretion [11]. Brisson et al. [43,44] demonstrated that a body-temperature threshold value had to be reached for heat stress to induce a significant blood Prl response during active or passive heat exposure. These authors estimated this mean T_rec _threshold to be approximately 1.3°C – 1.7°C above normal body temperature values. In the present experiment, the rise in T_rec _induced by exercise in the heat far exceeded this threshold in both pre- and post-supplementation trials, thus resulting in a similar elevation in plasma [Prl] in both Plc and Cr groups (Table 4). However, T_rec _was lower in the post-Cr supplementation trial and T_rec _and plasma [Prl] were not correlated. These results are in agreement with a number of previous studies [e.g., [45]] that showed that Prl secretion is not always related to plasma free-[Trp] and this may be more evident during exercise in the heat. Consequently, although Cr was effective in reducing thermal stress, this reduction was insufficient to significantly alter plasma [Prl]. However, this finding does not necessarily preclude a difference in hypothalamic Prl secretion between Plc and Cr trials.

A number of previous classic studies have reported a significant correlation between plasma [FFA] and plasma free-[Trp] primarily due to FFA displacing Trp from its binding to albumin [e.g., [46,47]]. In the present experiment, the rise in plasma [FFA] was modest and not different between trials but plasma free-[Trp] was lower during exercise and at exhaustion on the post-Cr supplementation trial. This somewhat surprising result, in conjunction with the lack of a significant correlation between plasma [FFA] and plasma free-[Trp] would suggest a more complex control of plasma free-[Trp] during exercise in the heat than previously described. Since differences in plasma volume, which could partially explain the reduction in plasma free-[Trp] and free-Trp:Tyr ratio were not observed in the present experiment, the exact mechanism(s) responsible for the observed reduction in plasma free-[Trp] and free-Trp:Tyr ratio after Cr loading (despite no difference in plasma [FFA]) and the significance of these observations remain to be determined.

The major limitations of the experiment were the indirect method used for classifying the participants into "responders" and "non-responders" to Cr. However, by not pre-distinguishing the potential "responders" and "non-responders" to each particular ergogenic aid supplement, such as Cr treatment, the type II statistical error might has been elevated. Syrotuik and Bell [48] for example, examined the physiological profile of "responders" and "non-responders" to 5-days Cr loading sub-divided based on their mean changes in resting muscle Cr + PCr levels. They found that there were three subdivisions of Cr supplementation: the "responders", the "quasi-responders" and the "non-responders" to Cr, subdivided based on their intramuscular Cr uptake [with mean changes in resting Cr + PCr of 29.5 mmol.kg^-1 ^dw (n = 3), 14.9 mmol.kg^-1 ^dw (n = 5), and 5.1 mmol.kg^-1 ^dw (n = 3), respectively]. They also observed that the "responders" had a higher percentage of type II muscle fibres and the greatest pre-load muscle fibres cross-sectional area and fat free mass. The same group showed an improvement in 1RM leg press score following the Cr loading period. Whereas, the "non-responders" group had exactly the opposite results than the responders group following the 5-days Cr loading period. It is plausible therefore that the lack of differences in exercise performance observed in the present experiment between the whole Cr and the Plc groups may be due to a person-by-treatment different physiological response to Cr supplementation. Furthermore, this may be applied in several other studies which examined central fatigue during prolonged exercise either in the heat or in thermoneutral environment where pharmacological treatment such as selective serotonin reuptake inhibitors (SSRI) and/or other precursors and inhibitors of brain serotonergic and dopaminergic systems were used as interventions. In a psychophysiological study for example, where the differences between the "responders" and "non-responders" to drug (brofaromine and fluvoxamine) therapy were examined, it was suggested that heart rate and blood pressure responsiveness to the drugs both were different between the two groups, higher in the "non-responders" than the "responders" to the drug [49]. Similarly, Kampf-Sherf et al. [50] examined the physiological responses to SSRI treatment to depressed patients and they suggested that only two third of patients with major depression has shown a physiological responses to antidepressants such as SSRI. Consequently, the present results may help partially explain the equivocal performance and other physiological responses results reported in the literature following ergogenic aid supplements and/or drug interventions of brain 5-HT and DA systems manipulation.

## Conclusion

In conclusion, the effectiveness of Cr to reduce thermal stress *per se *and/or alter key modulators of brain 5-HT and DA function may have contributed to the reduced effort perception during exercise in the heat but not to that extent in eliciting an enhancement of endurance performance in the whole Cr group. Consequently, the results presented here are consistent with a centrally-mediated process of fatigue during strenuous exercise in the heat either with or without using Cr supplementation. It is possible however, that Cr may improve performance only in the "responders" to Cr; however, more studies are warranted to justify this concept. The present results may also suggest the demanding of the pre-experimental physiological identification of the "responders" and "non-responders" to Cr treatment. Otherwise, the possibility of the type II statistical error may be augmented. Future studies need to investigate the direct effects of Cr supplementation on brain 5-HT and DA systems and their association with central fatigue development during exercise in the heat.

## Competing interests

The authors declare that they have no competing interests.

## Authors' contributions

MH was the primary author of the manuscript and participated in the design of the study and carried out the data collection, data analysis, statistical analysis and interpretation of the results. LK played an important role in study design, data collection, data analysis, statistical analysis and interpretation and manuscript preparation. YP played an important role in study design, data collection, data interpretation and study coordination. All authors read and approved the final manuscript.

## References

[B1] Nybo L (2007). Exercise and heat stress: cerebral challenges and consequences. Prog Brain Res.

[B2] Galloway SDR, Maughan RJ (1997). Effects of ambient temperature on the capacity to perform prolonged cycle exercise in man. Med Sci Sports Exerc.

[B3] Nybo L, Nielsen B (2001). Hyperthermia and central fatigue during prolonged exercise in humans. J Appl Physiol.

[B4] Nielsen B, Hales J, Strange S, Christensen N, Warberg J, Saltin B (1993). Human circulatory and thermoregulatory adaptations with heat acclimation and exercise in a hot, dry environment. J Physiol.

[B5] Nybo L, Nielsen B (2001). Middle cerebral artery blood velocity is reduced with hyperthermia during prolonged exercise in humans. J Physiol.

[B6] Nybo L, Moller K, Volianitis S, Nielsen B, Secher N (2002). Effects of hyperthermia on cerebral blood flow and metabolism during prolonged exercise in humans. J Appl Physiol.

[B7] Nielsen B, Nybo L (2003). Cerebral changes during exercise in the heat. Sports Med.

[B8] Nybo L, Nielsen B, Blomstrand E, Moller K, Secher N (2003). Neurohumoral responses during prolonged exercise in humans. J Appl Physiol.

[B9] Hasegawa H, Yazawa T, Yasumatsu M, Otokawa M, Aihara Y (2000). Alteration in dopamine metabolism in the thermoregulatory centre of exercising rats. Neurosci Lett.

[B10] Lin MT, Tsay HJ, Su WH, Chueh FY (1998). Changes in extracellular serotonin in rat hypothalamus affect thermoregulatory function. Am J Physiol.

[B11] Pitsiladis Y, Strachan A, Davidson I, Maughan R (2002). Hyperprolactinaemia during prolonged exercise in the heat: evidence for a centrally mediated component of fatigue in trained cyclists. Exp Physiol.

[B12] Soares DD, Lima NRV, Coimbra CC, Marubayashi U (2004). Intracerebroventricular tryptophan increases heating and heat storage rate in exercising rats. Pharmacol Biochem Behav.

[B13] Soares DD, Coimbra CC, Marubayashi U (2007). Tryptophan-induced central fatigue in exercising rats is related to serotonin content in preoptic area. Neurosci Lett.

[B14] Bridge MW, Weller AS, Rayson M, Jones DA (2003). Responses to exercise in the heat related to measures of hypothalamic serotonergic and dopaminergic function. Eur J Appl Physiol.

[B15] Watson P, Hasegawa H, Roelands B, Piacentini MF, Looverie R, Meeusen R (2005). Acute dopamine/noradrenaline reuptake inhibition enhances human exercise performance in warm, but not temperate conditions. J Physiol.

[B16] Watanabe A, Kato N, Kato T (2002). Effects of creatine on mental fatigue and cerebral hemoglobin oxygenation. Neurosci Res.

[B17] Harris RC, Soderlund K, Hultman E (1992). Elevation of creatine in resting and exercised muscle of normal participants by creatine supplementation. Clin Sci.

[B18] Greenhaff PL, Bodin K, Soderlund K, Hultman E (1994). Effect of oral Creatine supplementation on skeletal muscle phosphocreatine resynthesis. Am J Physiol.

[B19] Easton C, Turner S, Pitsiladis YP (2007). Creatine and glycerol hyperhydration in trained participants before exercise in the heat. Int J Sports Nutr Exerc Metab.

[B20] Hultman EK, Soderlund JA, Timmons G, Greenhaff PL (1996). Muscle creatine loading in men. J Appl Physiol.

[B21] Kern M, Podewils LJ, Vukovich M, Buono MJ (2001). Physiological response to exercise in the heat following creatine supplementation. J Exerc Physiol [online].

[B22] Klivenyi P, Gardian G, Calingasan Y, Yang L, Beal MF (2003). Additive neuroprodective effects of creatine and a cyclooxygenase 2 inhibitor against dopamine depletion in the 1-methyl4-phenyl-1,2,3,6-tetrahydropyridine (MPTP) mouse model of Parkinson's disease. J Mol.

[B23] Klivenyi P, Ferrante RJ, Matthews RT, Bogdanov MB, Klein AM, Andreassen OA, Mueller G, Wermer M, Kaddurah-Daouk R, Beal MF (1999). Neuroprotective effects of creatine in a transgenic animal model of amyotrophic lateral sclerosis. Nature Med.

[B24] Matthews R, Ferrante R, Klivenyi P, Yang L, Klein A, Mueller G, Kaddurah-Daouk R, Beal M (1999). Creatine and cyclocreatine attenuate MPTP neurotoxicity. Exp Neurol.

[B25] Forster V, Dempsey J, Thomson J, Vidruk R, DoPico G (1972). Estimation of arterial PO_2_, PCO_2_, pH and lactate from arterialised venous blood. J Appl Physiol.

[B26] Dill DB, Costill DL (1974). Calculation of percentage changes in volumes of blood, plasma, and red cells in dehydration. J Appl Physiol.

[B27] Ramanthan NL (1964). A new weighting system for mean surface temperature of the human body. J Appl Physiol.

[B28] Olschewski H, Bruk K (1988). Thermoregulatory, cardiovascular, and muscular factors related to exercise after precooling. J Appl Physiol.

[B29] Kilduff LP, Vidakovic P, Cooney G, Twycross-Lewis R, Amuna P, Parker M, Paul L, Pitsiladis YP (2002). Effects of creatine on isometric bench-press performance in resistance-trained humans. Med Sci Sports Exerc.

[B30] Casey A, Contantin-Teodosiu D, Howell S, Hultman E, Greenhaff PL (1996). Creatine ingestion favourably affects performance and muscle metabolism during maximal exercise in humans. Am J Physiol.

[B31] Bergstrom J, Guarnieri G, Hultman E (1971). Carbohydrate metabolism and electrolyte changes in muscle tissue during heavy work. J Appl Physiol.

[B32] Volek JS, Mazzetti SA, Farquhar WB, Barnes BR, Gomez AL, Kraemer WJ (2001). Physiological responses to short-term exercise in the heat after creatine loading. Med Sci Sports Exerc.

[B33] Davis J, Bailey S (1997). Possible mechanisms of central nervous system fatigue during exercise. Med Sci Sports Exerc.

[B34] Brown SJ, Gisolfi CV, Mora F (1982). Temperature regulation and dopaminergic systems in the brain: does the substantia nigra play a role. Brain Res.

[B35] Cox B, Lee TF (1980). Further evidence for a physiological role for hypothalamic dopamine in thermoregulation in the rat. J Physiol.

[B36] Lieberman HR, Georgelis JH, Maher TJ, Yeghiaya SK (2005). Tyrosine prevents effects of hyperthermia on behaviour and increases norepinephrine. Physiol Behav.

[B37] Newsholme E, Acworth IN, Blomstrand E, Benzi G (1987). Amino-acids, brain neurotransmitters and a functional link between muscle and brain that is important in sustained exercise. Advances in Biochemistry.

[B38] Bloxam DL, Tricklebank M, Patel A, Curzon G (1980). Effects of albumin amino acids and clofibrate on the uptake of tryptophan by the rat brain. J Neurochem.

[B39] Fernstrom JD, Wurtman RJ (1972). Brain serotonin content: physiological regulation by plasma neutral amino acids. Science.

[B40] Wurtman R (1988). Effects of their nutrient precursors on the synthesis and release of serotonin, the catecholamine, and acetylcholine: implications for behavioural disorders. Clin Neuropharmacol.

[B41] Leathwood PD, Fernstrom JD (1990). Effect of an oral tryptophan/carbohydrate load on tryptophan, large neutral amino acid, and serotonin and 5-hydroxyindoleacetic acid levels in monkey brain. J Neural Transm Gen Sect.

[B42] Fischer HG, Hollmann W, De Meirleir K (1991). Exercise changes in plasma tryptophan fractions and relationship with prolactin. Int J Sports Med.

[B43] Brisson G, Peronnet R, Ledoux M, Pellerin-Massicotte J, Matton P, Garceau F, Boisvert JrP (1986). Temperature-induced hyperprolactinemia during exercise. Horm Metab Res.

[B44] Brisson G, Peronnet R, Perrault H, Boisvert P, Massicotte D, Gareau R (1991). Prolactinotrophic effect of endogenous and exogenous heat loads in human male adults. J Appl Physiol.

[B45] Struder H, Hollmann W, Platen P, Duperly J, Fischer H, Weber K (1996). Alterations in plasma free tryptophan and large neutral amino acids do not affect perceived exertion and prolactin during 90 min of treadmill exercise. Int J Sports Med.

[B46] Curzon G, Friedel J, Knott PJ (1973). The effect of fatty acids on the binding of tryptophan to plasma protein. Nature.

[B47] Spector AA (1975). Fatty acid binding to plasma albumin. J Lipid Res.

[B48] Syrotuik DG, Bell GJ (2004). Acute creatine monohydrate supplementation: a descriptive physiological profile of responders vs nonresponders. J Strength Cond Res.

[B49] Slaap BR, van Vliet IM, Westenberg HGM, Den Boer JA (1996). Responders and non-responders to drug treatment in social phobia: differences at baseline and prediction of response. J Affect Disord.

[B50] Kampf-Sherf O, Zlotogorski Z, Gilboa A, Speedie L, Lereya J, Rosca P, Shavit Y (2004). Neuropsychological functioning in major depression and responsiveness to selective serotonin reuptake inhibitors antidepressants. J Affect Disord.

[B51] Kilduff LP, Georgiades E, James N, Minnion RH, Mitchell M, Kingsmore D, Hadjicharlambous M, Pitsiladis YP (2004). The effects of creatine supplementation on cardiovascular, metabolic, and thermoregulatory responses during exercise in the heat in endurance-trained humans. Int J Sport Nutr Exerc Metab.

